# Purification and Characterization of Novel Collagen Peptides from *Oncorhynchus mykiss* Inhibiting Platelet Aggregation, and the Underlying Mechanism via Molecular Docking

**DOI:** 10.3390/foods15142507

**Published:** 2026-07-15

**Authors:** Haodong Liu, Linjing Zhang, Zhiyong Zeng, Xin Wang, Bo Li

**Affiliations:** 1 College of Food Science and Nutritional Engineering, China Agricultural University, Beijing 100083, China; liuhaodong@cau.edu.cn (H.L.); zhanglinjing0418@163.com (L.Z.); 19979482776@163.com (Z.Z.); 15725655790@163.com (X.W.); 2 Key Laboratory of Functional Dairy, Ministry of Education, Beijing 100083, China

**Keywords:** rainbow trout, bioactive peptides, platelet aggregation inhibition, P2Y12 receptor, molecular docking

## Abstract

Rainbow trout is produced in large quantities worldwide, generating substantial processing by-products, among which collagen-rich skin and bone require high-value utilization. Rainbow trout collagen peptides potentially exhibit antiplatelet activity based on structural similarity to reported antiplatelet fish collagen peptides, while their active peptide sequences and underlying mechanisms require investigation. In this study, rainbow trout skin collagen was hydrolyzed by neutral protease, yielding a hydrolysate with 70.52% inhibition of platelet aggregation at 4 mg/mL. The fraction with high antiplatelet activity, N2-2, was obtained via separation by ODS-C18 and Sephadex LH-20 chromatography, which was enriched in Gly, Pro, and Hyp residues and characterized by mild hydrophilicity and a moderate molecular weight. Peptide sequences in the N2-2 were identified by HPLC-MS/MS, followed by molecular docking-based screening, and the four peptides selected were synthesized and evaluated for antiplatelet activity in vitro. Peptides MTGP and OOGGHG exhibited higher antiplatelet activity, with IC_50_ values of 0.52 mM and 0.54 mM, respectively. Molecular docking indicated that MTGP and OOGGHG stably bind to the P2Y12 receptor via hydrogen bonding with key residues (Lys280, Arg256, and Asn191), with hydrophobic interactions contributing to complex stability. This study adds support to the feasibility of natural antiplatelet functional foods from rainbow trout collagen.

## 1. Introduction

Rainbow Trout (*Oncorhynchus mykiss*) is one of the most important cold-water aquaculture species worldwide. It is characterized by rapid growth, high feed conversion efficiency, and desirable flesh quality, occupying a central position in global cold-water aquaculture. According to the latest statistics from the Food and Agriculture Organization of the United Nations, global production of rainbow trout exceeded 1.02 million tons in 2024 [1]. As the largest producer of rainbow trout in Asia, China produces approximately 100,000 tons annually, accounting for about 10% of global production, with rainbow trout farming mainly concentrated in Gansu, Qinghai, Xinjiang, Shandong, and Beijing [2]. At present, rainbow trout is conventionally consumed in the form of chilled or frozen fillets, smoked products, and sashimi; however, by-products generated under these processing conditions account for approximately 50–60% of the total fish mass [3]. Fish skin and bones are generally regarded as low-value by-products and are mainly used for the production of pet feed or fertilizer, while their potential value has not yet been fully exploited [4].

Rainbow trout skin and scales contain abundant collagen (accounting for approximately 70% of the dry weight of the skin) and are ideal raw materials for the preparation of bioactive peptides [5]. Collagen is rich in glycine (Gly), proline (Pro), and hydroxyproline (Hyp), with Gly accounting for approximately 30% of the total amino acid content, and exhibits the typical Gly-X-Y repeating sequence characteristic of collagen [6]. The collagen peptides enriched in Gly, Pro, and Hyp residues have been reported to exhibit antioxidant activity and angiotensin-converting enzyme (ACE) inhibitory activity [7,8,9]. In addition to these biological functions, antithrombotic bioactive peptides derived from aquatic processing by-products have attracted attention in recent years. Collagen peptides derived from Atlantic salmon (*Salmo salar*) skin and silver carp (*Hypophthalmichthys molitrix*) skin containing Hyp-Gly or Pro-Gly sequences have been reported to significantly inhibit adenosine diphosphate (ADP)-induced platelet aggregation, which implies similar antithrombotic potential in rainbow trout collagen peptides [10,11]. Nevertheless, direct evidence for its antiplatelet activity requires further investigation.

With the progression of population aging, the number of individuals at risk of cardiovascular diseases (CVDs) continues to increase. Aging-associated increases in circulating ADP levels, platelet hyperreactivity, and endothelial dysfunction collectively promote a pro-thrombotic state, thereby increasing the risk of atherosclerosis and acute cardiovascular events [12]. However, synthetic antiplatelet drugs, such as aspirin, prasugrel, and clopidogrel, are mainly used for the treatment of diagnosed thrombotic diseases and are not appropriate for long-term preventive use in at-risk populations [13]. Therefore, dietary intervention with safe and effective bioactive peptides has emerged as a promising strategy for the prevention and control of thrombotic diseases [14].

The purinergic receptor P2Y12 is a key receptor involved in ADP-mediated platelet activation and signal amplification. Activation of P2Y12 inhibits adenylate cyclase through Gi protein signaling, reduces intracellular cAMP levels, and promotes the PI3K/Akt pathway and GPIIb/IIIa integrin activation, thereby enhancing platelet aggregation and stabilizing thrombus formation [15,16]. Therefore, the platelet P2Y12 receptor is considered an important target for the prevention of thrombosis and related CVDs, and elucidating the interactions between bioactive peptides and the P2Y12 receptor is of great significance for understanding their antiplatelet mechanisms.

In this context, this study aimed to identify antiplatelet peptides derived from rainbow trout skin and to investigate their potential interactions with the P2Y12 receptor. To achieve this, the antiplatelet peptides were prepared from rainbow trout skin collagen via enzymatic hydrolysis, followed by separation, purification, and identification. Subsequently, molecular docking was performed to screen candidate peptides with high binding affinity to the P2Y12 receptor, and their activities were further verified in vitro. Furthermore, the interaction patterns and key binding sites between these peptides and the P2Y12 receptor were elucidated.

## 2. Materials and Methods

### 2.1. Materials and Reagents

Fresh frozen rainbow trout skin was used as the raw material obtained from Fuyang Seafood Store (Qingdao, China). Neutral protease (20,000 U/g) was purchased from Pangbo Enzyme Co., Ltd. (Nanning, China). ADP (Cat. No. A0180, CAS 58-64-0) and aspirin (Cat. No. A8830, CAS 50-78-2) were purchased from Solarbio Science & Technology Co., Ltd. (Beijing, China). All other chemicals and reagents utilized were of analytical grade.

### 2.2. Extraction of Collagen from Rainbow Trout Skin

The fish skin was washed with water, and the scales, subcutaneous fat, and residual muscle were removed. The cleaned skin was cut into ~0.5 cm^2^ pieces and treated with 0.05 mol/L NaOH solution (1:6, *w*/*v*) for 1 h, then rinsed to neutral. Subsequently, the skin was soaked in 0.2% H_2_SO_4_ (1:6, *w*/*v*) for 1 h and rinsed again. The pretreated skin was extracted with distilled water (1:3, *w*/*v*) at boiling temperature for 3 h. The extract was filtered, and the supernatant was freeze-dried to obtain collagen.

### 2.3. Preparation of Collagen Hydrolysates

Collagen was mixed with deionized water (8.0%, *w*/*v*) and adjusted to pH 7.0 with 1 mol/L NaOH. Enzymatic hydrolysis was carried out at 55 °C with neutral protease addition levels of 0.8%, 1.0%, and 1.5% (*w*/*w*) for 1.0–4.0 h. The reaction was terminated by boiling for 10 min. The hydrolysate was centrifuged (10,000× *g*, 20 min), and the supernatant was collected, freeze-dried, and used for the antiplatelet activity assay.

### 2.4. Determination of Degree of Hydrolysis (DH)

DH was determined using the o-phthaldialdehyde (OPA) method [17]. The OPA reagent was prepared by dissolving sodium tetraborate (3.810 g) and SDS (100 mg) in 75 mL of deionized water, followed by the addition of OPA (80 mg in 2 mL ethanol) and DTT (88 mg), and dilution to 100 mL. A sample solution at 1.0 mg/mL was mixed with OPA reagent in a ratio of 100 μL to 1 mL and reacted for 2 min. Absorbance was measured at 340 nm. Serine was used as the standard. DH was calculated as follows:

$$DH\left(\%\right)=\frac{N-N_{0}}{C\times h_{tot}}\times 100\%$$

where N is the amino nitrogen content of the hydrolysate, N_0_ is the amino nitrogen content of the collagen before hydrolysis, C is the protein concentration, and h_tot_ is the total number of peptide bonds in collagen, taken as 8.0 mmol/g protein according to previous studies [18,19].

### 2.5. In Vitro Antiplatelet Activity Measurement

#### 2.5.1. Preparation of Platelet-Rich Plasma (PRP)

Eight-week-old male Sprague Dawley (SD) rats (220–250 g) were obtained from Beijing Vital River Laboratory Animal Technology Co., Ltd. (Beijing, China). Blood was collected from rats anesthetized with Avertin using sodium citrate as an anticoagulant. The blood was diluted with PBS (1:1, *v*/*v*) and centrifuged (50× *g*, 10 min) to obtain PRP. The remaining fraction was further centrifuged (750× *g*, 10 min) to obtain platelet-poor plasma (PPP). Platelet concentration in PRP was adjusted to 2–3 × 10^8^ platelets/mL and used within 2 h.

#### 2.5.2. Antiplatelet Activity Assay

A total of 270 μL of PRP was mixed with 30 μL of each sample (collagen hydrolysates, synthesized peptides, or aspirin) in test cuvettes. The mixtures were incubated at 37 °C for 5 min in the preheating wells of an AG400 fully automated platelet aggregation analyzer (Techlink Biomedical Technology Co., Ltd., Beijing, China). The cuvettes were then transferred to the detection channels, followed by the addition of ADP (final concentration, 100 μM) to induce platelet aggregation. Platelet aggregation was measured after 5 min, and the antiplatelet activity of each sample was calculated using the following equation:

$$Antiplatelet\;Activity\left(\%\right)=\frac{ARC-ARS}{ARC}\times 100\%$$

where ARC and ARS represent the aggregation rate of the control group and sample group, respectively.

### 2.6. Reverse-Phase C18 Fractionation

Collagen hydrolysates from rainbow trout skin were dissolved in water (90 mg/mL) and filtered through a 0.45 μm membrane. A 1 mL aliquot was loaded onto an ODS-A-HG reverse-phase C18 packing material (YMC Co., Japan; Φ1.0 cm × 10 cm, bed volume 7 mL). Elution was performed at 1 mL/min using a stepwise gradient of deionized water (0–13 min), 10% methanol (13–26 min), 30% methanol (26–40 min), and 50% methanol (40–60 min), with detection at 220 nm. Fractions were collected based on chromatographic peaks, concentrated by rotary evaporation, freeze-dried, and stored at −80 °C for further analysis.

### 2.7. Sephadex LH-20 Gel Filtration Fractionation

C18-derived peptide fractions were dissolved in water (200 mg/mL) and filtered through a 0.45 μm membrane. A 3 mL aliquot was loaded onto Sephadex LH-20 gel filtration packing material (Cytiva, Danaher Corporation, Washington, DC, USA; bed volume 100 mL; Φ4.0 cm × 60 cm). Elution was performed with 60% ethanol at 1 mL/min, with detection at 220 nm. Fractions were collected according to chromatographic peaks, concentrated by rotary evaporation, freeze-dried, and stored at −80 °C for further analysis.

### 2.8. Amino Acid Composition Analysis

Amino acid composition was determined by pre-column derivatization HPLC [20]. Samples (6 mg) were hydrolyzed with 6 M HCl at 110 °C for 24 h, evaporated to dryness, and re-dissolved in 0.1 M HCl. Derivatization was performed using phenylisothiocyanate (PITC) and triethylamine, followed by extraction and filtration (0.22 μm). Analysis was conducted on the LC-15C system (Shimadzu Corporation, Kyoto, Japan) equipped with an AdvanceBio AAA column (4.6 mm × 100 mm, 2.7 μm, Agilent Technologies, Santa Clara, CA, USA) at 254 nm with a flow rate of 1 mL/min. A phosphate–borate buffer (pH 8.2) and acetonitrile–methanol–water (45:45:10, *v*/*v*/*v*) system were used as mobile phases with gradient elution. Amino acid contents were quantified using standard calibration curves.

### 2.9. nanoLC-MS/MS

Samples were analyzed using an Orbitrap Fusion Lumos mass spectrometer (Thermo Fisher Scientific, Waltham, MA, USA) coupled with an EASY-nanoLC 1200 system (Thermo Fisher Scientific, Waltham, MA, USA). Peptides were separated on a C18 column (20 cm × 75 μm, 1.9 μm) (Thermo Fisher Scientific, Waltham, MA, USA) with a 60 min gradient (4–50% acetonitrile with 0.1% formic acid) at 300 nL/min and 40 °C. MS data were acquired in the *m*/*z* 100–1500 range at 120,000 resolution. MS/MS data were matched against the UniProt database (https://www.uniprot.org/) using *Oncorhynchus mykiss* skin collagen sequences as a reference.

### 2.10. Molecular Docking Analysis

Molecular docking was performed to analyze the interaction between active peptides and the P2Y12 receptor [21]. Peptide structures were generated from amino acid sequences using ChemBioDraw and optimized in ChemBio3D (ChemOffice Ultra 14.0). The crystal structure of P2Y12 (PDB ID: 4PXZ) was obtained from the RCSB Protein Data Bank and prepared using PyMOL 2.6.2 (Schrödinger, LLC, New York, NY, USA) by removing water molecules and co-crystallized ligands and adding polar hydrogens. Docking was carried out using AutoDock Vina 1.2.0 with grid center coordinates of x = 16.911, y = 2.681, and z = 54.896 [21]. Binding interactions were analyzed and visualized using PyMOL 2.6.2 (Schrödinger, LLC, New York, NY, USA) and Discovery Studio 2021 (BIOVIA, Dassault Systèmes, Waltham, MA, USA).

### 2.11. Chemical Synthesis of Peptides

The candidate peptides were chemically synthesized by SYNPEPTIDE Co., Ltd. (Nanjing, China) using the standard Fmoc-based solid-phase peptide synthesis method. The synthesized peptides were converted to the acetate salt form, and the purity of all peptides was >98%.

### 2.12. Statistical Analysis

Data were analyzed and plotted using GraphPad Prism 10.2.0 (GraphPad Software, San Diego, CA, USA). Results are presented as mean ± standard deviation (SD) from at least three independent experiments. Statistical differences were evaluated by one-way analysis of variance (ANOVA), with *p* < 0.05 considered statistically significant.

## 3. Results and Discussion

### 3.1. Evaluation of the Antiplatelet Activity of Collagen Hydrolysates Under Different Enzymatic Hydrolysis Conditions

**Figure 1:**
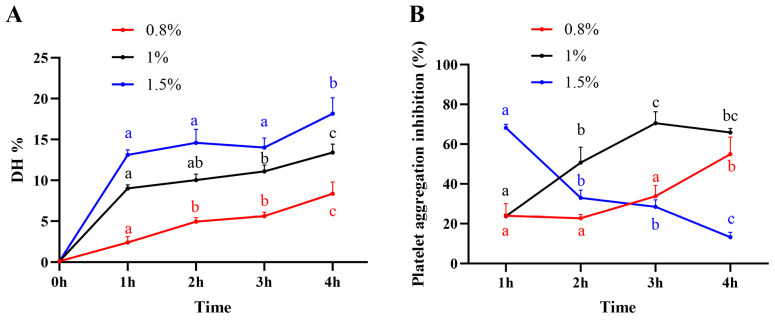
Effects of enzyme dosage and hydrolysis time on the degree of hydrolysis (**A**) and antiplatelet activity (**B**) of rainbow trout fish skin collagen hydrolysates prepared with neutral protease. Data are expressed as mean ± standard deviation (*n* = 3). Note: Different lowercase letters above data points of the same color line indicate significant differences among the hydrolysates under the same enzyme–substrate ratio (*p* < 0.05).

As shown in Figure 1A, the DH of the collagen increased significantly with increasing enzyme dosage and prolonged hydrolysis time. Within one hour, the DH increased the fastest, and the larger the amount of enzyme used, the higher the DH. Specifically, the DH significantly increased to 2.41%, 9.01%, and 13.11% in 1 h at the corresponding enzyme dosages of 0.8%, 1.0%, and 1.5%, respectively. As the enzymatic hydrolysis time increased, the DH continued to increase. The 1.5% enzyme dosage group exhibited the highest hydrolytic efficiency, further significantly increasing to 18.16% at 4 h (*p* < 0.05).

The antiplatelet activity of the hydrolysates prepared under each hydrolysis condition was evaluated at 4 mg/mL, and the results are presented in Figure 1B. In the 0.8% enzyme dosage group, the inhibition rate increased continuously with hydrolysis time, from 23.68% at 1 h to 54.95% at 4 h, with the activity at 4 h being significantly higher than that at 1–3 h (*p* < 0.05). In the 1.0% enzyme dosage group, the inhibition rate first increased and then decreased, rising significantly from 23.77% at 1 h to a peak of 70.52% at 3 h (*p* < 0.05), followed by a slight decrease to 65.90% at 4 h, while still maintaining relatively high activity. In contrast, the 1.5% enzyme dosage group reached its maximum inhibition rate of 68.24% at 1 h, after which the activity decreased significantly with prolonged hydrolysis time (*p* < 0.05), dropping to 13.18% at 4 h.

The results show that the antiplatelet activity is related to the DH of the hydrolysate, and controlling the DH within an appropriate range of 9–15% can yield highly antiplatelet peptides. Although a high enzyme dosage (1.5%) yielded a hydrolysis degree of 13.11% within 1 h and achieved relatively high antiplatelet activity, the increased enzyme usage would lead to higher production costs. Therefore, an enzyme dosage of 1.0% and a hydrolysis time of 3 h were selected as the optimal conditions for subsequent preparation, under which the hydrolysate exhibited an inhibition rate of 70.52% at 4 mg/mL, markedly higher than those reported for salmon skin hydrolysates prepared using Alcalase (40.40%) or Protamex (35.63%) [10], and silver carp skin hydrolysates (~37%) at the same concentration [11], indicating the potential of rainbow trout skin hydrolysates as functional antiplatelet ingredients in food applications.

### 3.2. Separation of Antiplatelet Peptides

**Figure 2:**
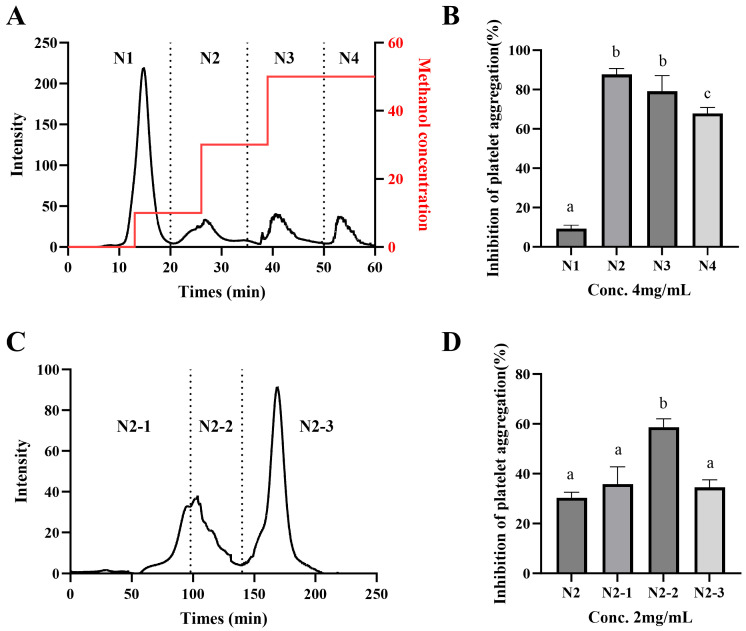
Fractionation of collagen hydrolysates by ODS-C18 column chromatography (**A**) and the antiplatelet activity of the resulting fractions (**B**); secondary fractionation of fraction N2 by Sephadex LH-20 gel filtration chromatography (**C**) and the antiplatelet activity of the resulting subfractions (**D**). Data are expressed as mean ± standard deviation (*n* = 3). Note: Different lowercase letters indicate significant differences among groups (*p* < 0.05).

Neutral protease hydrolysates were further separated via an ODS-C18 column to analyze the elution behavior and antiplatelet activity of individual fractions (Figure 2A). Under a methanol–water gradient system, the hydrolysates were well separated into four distinct fractions (N1–N4). N1 dominated in the 0–10% low-methanol elution phase, whereas N2, N3, and N4 were sequentially eluted with increasing methanol content and displayed comparable peak abundances. The antiplatelet activity of the separated fractions (Figure 2B) showed that N1 exhibited the weakest inhibitory activity, with a platelet aggregation inhibition rate of only 9.18% at a concentration of 4 mg/mL, significantly lower than that of N2, N3, and N4 (*p* < 0.05). N2 exhibited the highest inhibitory activity, with a rate of 87.61%, followed by N3 (79.11%) and N4 (67.77%).

The N2 fraction with the highest bioactivity was further purified using Sephadex LH-20 based on molecular size exclusion. As shown in Figure 2C, high-molecular-weight peptides (N2-1) were eluted first, followed by medium-sized (N2-2) and small-sized peptides (N2-3). The antiplatelet activity of these fractions (Figure 2D) showed that at a concentration of 2 mg/mL, N2-2 exhibited an inhibition rate of 58.71%, significantly higher than that of N2-1 (35.81%) and N2-3 (34.45%) (*p* < 0.05).

The ODS-C18 separation results indicate that the peptides with higher antiplatelet activity are primarily eluted at 10–30% methanol (N2). Similarly, in a previous study using the collagen hydrolysate derived from Atlantic salmon skin separated by ODS-C18, the F2 fraction (eluted with 10% methanol) showed the highest antiplatelet activity, with an inhibition rate of 88.52% at 4 mg/mL [10]. After separation of collagen hydrolysate derived from white amur fish skin by the macroporous resin XAD-16, the 20% ethanol elution fraction exhibited the strongest antiplatelet activity, with an IC_50_ of 2.03 mg/mL [11]. The results obtained in this study regarding the elution polarity ranges of the active components are consistent with the results reported by previous authors.

The results of Sephadex LH-20 separation indicate that the peptides with a medium-molecular-weight range may be responsible for the antiplatelet activity, which aligns with the appropriate DH (Figure 1 and Figure 2). The antiplatelet activity of peptides is related to the degree of hydrolysis or the molecular weight range. The N2-1 fraction, due to its large molecular weight, was less capable of binding to target proteins, resulting in limited activity, while the small peptides in N2-3 may lack complete functional sequences, leading to a significant reduction in their antiplatelet ability.

### 3.3. Amino Acid Composition of the Antiplatelet Peptides

The amino acid compositions of native rainbow trout skin collagen, collagen hydrolysates, and the N2-2 fraction were analyzed (Table 1). Native skin collagen presented the typical collagen amino acid profile, with glycine dominating (accounting for nearly one-third of total amino acids) and corresponding to the conserved Gly-X-Y repeating sequence, whose content falls within the reported range of 24.16–36.90 g/100 g. Proline was also abundant at 9.71 g/100 g, in accordance with previous findings of 9.50–11.10% [22,23,24]. Compared with native collagen, the hydrolysates showed no obvious alterations in amino acid composition, with slight elevations in Gly (30.84 g/100 g), Pro (10.31 g/100 g), and Hyp (6.90 g/100 g).

Notably, further purification altered the amino acid profile of the N2-2 fraction. N2-2 exhibited substantial increases in Gly (33.64 g/100 g), Hyp (13.69 g/100 g), and Pro (15.75 g/100 g), with the combined content of these three amino acids reaching approximately 60 g/100 g. This result suggests that the N2-2 fraction is enriched in Gly, Pro, and Hyp, which are typically derived from the collagen triple helix region and are crucial for maintaining structural stability [25]. The total hydrophilic amino acid content in N2-2 increased to 56.21 g/100 g, compared with 47.07 g/100 g in the hydrolysates, which is consistent with the selective nature of the separation system. The amino acid composition suggests that N2-2 possesses a mild hydrophilic characteristic, which may be related to its antiplatelet activity.

### 3.4. Identification of Peptide Sequences from N2-2

**Figure 3:**
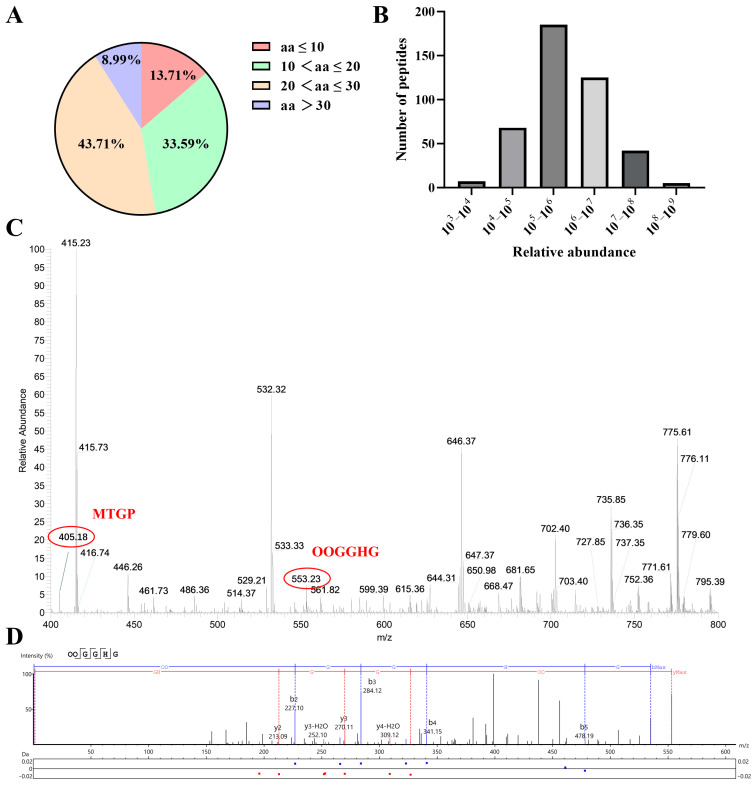
Characterization of peptide distribution and mass spectrometry analysis of N2-2: (**A**) The relative abundance distribution of peptides with different amino acid lengths. (**B**) Distribution of peptide counts across different abundance intervals. (**C**) Representative full-scan MS spectrum of peptides MTGP and OOGGHG. (**D**) MS/MS spectrum of the peptide OOGGHG. Note: In panel (**D**), blue lines indicate b ions and red lines indicate y ions. Blue and red dots at the bottom represent the mass errors of the identified b and y ions of OOGGHG, respectively.

A total of 553 peptides were identified in the N2-2 fraction using nanoLC–MS/MS analysis. Among them (Figure 3A), 92 oligopeptides (<10 amino acid residues) were identified, accounting for 13.71% of the total peptide abundance, whereas peptides with lengths of 10–20 and 20–30 amino acid residues represented the major fractions, comprising 33.59% and 43.71% of the total abundance, respectively. Peptide abundance analysis further revealed that most peptides were distributed within the 10^5^–10^6^ and 10^6^–10^7^ intensity ranges, with 185 and 125 peptides, respectively (Figure 3B). Among them, the information of the top 20 oligopeptides sorted by abundance was presented in Table 2. All peptide sequences were annotated through sequence alignment against the UniProt database, and the 20 identified peptides were mainly derived from the collagen type I alpha 1 chain (Collagen, type I, alpha 1a/1b), while a small number originated from fibrillar collagen NC1 domain-containing proteins, the collagen type XVII chain, and other homologous proteins of the collagen family.

Figure 3C illustrates the primary mass spectra at a retention time of 10–15 min, containing peptides MTGP and OOGGHG. Figure 3D illustrates the interpretation of the MS/MS spectra using peptide OOGGHG as an example. The parent ion at *m*/*z* 553.23 was identified as the peptide OOGGHG via calculation based on y-ions and b-ions and matched with the protein database derived from *Oncorhynchus mykiss* in UniProt. Other peptides were identified following the same method. Sequence analysis (Table 2) showed that the N2-2 peptides were rich in the typical collagen Gly–X–Y repeating motif, with frequent Gly, Pro, and Hyp residues, which agreed well with the amino acid composition results.

### 3.5. Antiplatelet Peptide Screening In Silico and Activity Validation

To further screen candidate peptides, molecular docking was employed as an in silico approach to evaluate their binding energy with the P2Y12 receptor. Considering that the oligopeptides have better biological accessibility than polypeptides and small-molecule peptides have simple structures and are easy to perform structural optimization calculations on [26], these highly abundant oligopeptides were subjected to molecular docking with the P2Y12 receptor to assess the binding interactions between the identified peptides and the P2Y12 receptor (Table 2). A binding energy below −6.0 kcal/mol indicates strong ligand–receptor binding [27]. Accordingly, four peptides with high binding affinity toward P2Y12, including three hexapeptides, namely LEGNMG (−7.1 kcal/mol), ANGPMG (−8.4 kcal/mol), and OOGGHG (−8.2 kcal/mol), and a tetrapeptide MTGP (−6.8 kcal/mol), were chemically synthesized for the verification test of antiplatelet activity.

**Figure 4:**
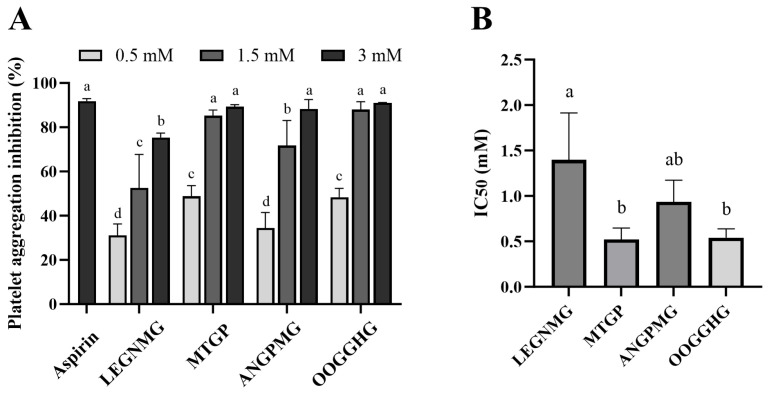
Antiplatelet activity of LEGNMG, MTGP, ANGPMG, and OOGGHG: (**A**) platelet aggregation inhibition rates of the four peptides at concentrations of 0.5 mM, 1.5 mM, and 3 mM, with aspirin (3 mM) as the positive control; (**B**) corresponding IC_50_ values of the four peptides. Data are expressed as mean ± standard deviation (*n* = 3). Note: Different lowercase letters indicate significant differences among groups (*p* < 0.05).

As shown in Figure 4A, aspirin (3 mM), which was used as a positive control, exhibited a high platelet aggregation inhibition rate of 91.8%. All tested peptides exerted inhibitory effects in a clear dose-dependent manner. In terms of antiplatelet activity, the peptides were ranked in descending order as follows: OOGGHG ≈ MTGP > ANGPMG > LEGNMG. The platelet aggregation inhibition rates of tetrapeptide MTGP and hexapeptide OOGGHG increased from 48.7% and 48.3% at 0.5 mM to 85.2% and 87.9% at 1.5 mM, respectively. A further increase in concentration to 3.0 mM resulted in only marginal improvements, reaching 89.3% and 91.1%, respectively, indicating that the inhibitory effects were approaching saturation (Figure 4A). Moreover, compared with the other two peptides, MTGP and OOGGHG exhibited significantly lower IC_50_ values of 0.52 mM and 0.54 mM, respectively (Figure 4B). The IC_50_ values of most identified collagen-derived peptides reported in the literature are between 0.2 and 5 mM [28,29]. In comparison, MTGP and OOGGHG have relatively high antiplatelet activities.

Overall, the antiplatelet activities of these peptides were consistent with the molecular docking results, with peptides exhibiting higher absolute binding energies tending to show stronger antiplatelet activity. However, tetrapeptide MTGP exhibits higher antiplatelet activity than the hexapeptides despite its less favorable binding energy. This discrepancy may arise from the inherent size bias of the scoring function employed by AutoDock Vina. Docking scoring functions often underestimate the binding affinity of small ligands, as they provide fewer atoms available for favorable non-covalent interactions, resulting in binding energies with smaller absolute values [27,30]. Consequently, the binding affinity of the tetrapeptide MTGP may be underestimated by molecular docking, resulting in a relatively unfavorable docking score despite its high antiplatelet activity.

Peptides with distinct structural features exhibited significant differences in antiplatelet activity, with peptides OOGGHG and MTGP showing the highest potency, likely due to their specific amino acid sequences. OOGGHG contains the characteristic Hyp-Gly (OG) motif, which has been identified as a key functional unit for antiplatelet activity in fish skin collagen peptides [17]. Similarly, the salmon-derived peptide OGEFG, harboring the OG motif, showed potent antiplatelet activity in ADP-induced platelet aggregation with an IC_50_ of 277.2 μM [5]. The Gly-Pro (GP) motif is also critical for antiplatelet activity; the collagen-derived tripeptide PGP inhibits thrombosis via anticoagulant effects, while salmon peptides WGPR and DEGP exert strong inhibition, with IC_50_ values of 208.7 μM and 290.0 μM, respectively [10,31]. The presence of the GP motif in MTGP and ANGPMG further supports the importance of the GP motif in antiplatelet activity. In contrast, the hexapeptide LEGNMG, without OG and GP motifs, exhibited relatively weak antiplatelet activity. These results further confirm OG and GP motifs as key structural features associated with the antiplatelet activity of collagen-derived peptides.

### 3.6. Molecular Docking Visualization of OOGGHG and MTGP

**Figure 5:**
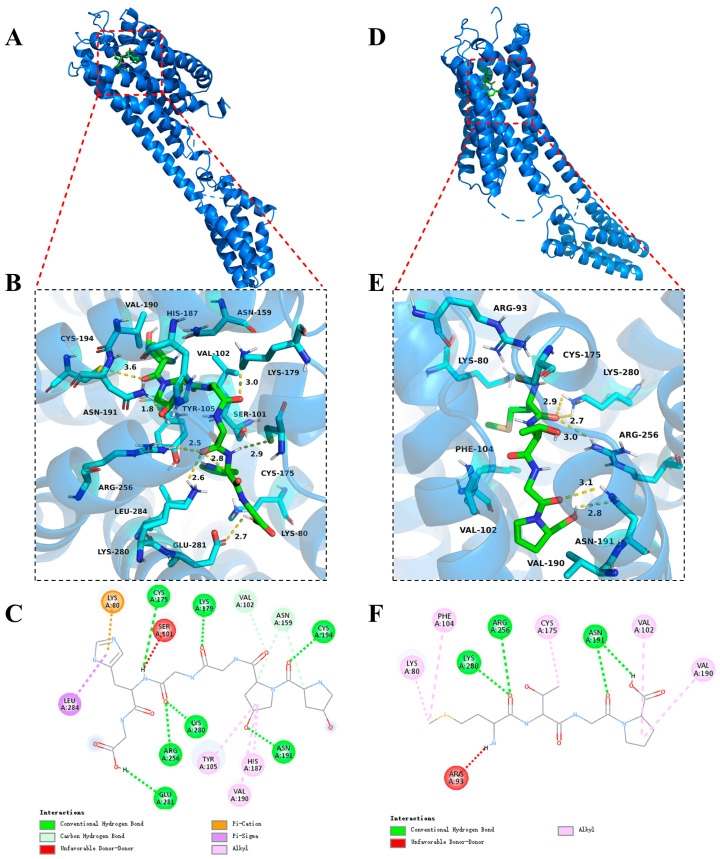
Molecular docking analysis of OOGGHG and MTGP with the P2Y12 receptor: (**A**–**C**) OOGGHG: overall binding mode (**A**), 3D interaction at the binding site (**B**), and 2D interaction map (**C**). (**D**–**F**) MTGP: overall binding mode (**D**), 3D interaction at the binding site (**E**), and 2D interaction map (**F**). Note: Blue sticks represent amino acid residues of P2Y12, and green sticks represent amino acid residues of OOGGHG (**B**) or MTGP (**E**). In panels (**C**,**F**), color-coded dashed lines represent different interaction types: dark green, conventional hydrogen bonds; light green, carbon–hydrogen bonds; pink, alkyl-type hydrophobic interactions; purple, π–σ interactions; orange, π–cation interactions; and red, unfavorable donor–donor interactions.

To further explore the potential binding mode of antiplatelet peptides with the P2Y12 receptor, OOGGHG and MTGP were selected as representative peptides for molecular docking visualization (Figure 5). OOGGHG was predicted to form seven conventional hydrogen bonds with polar residues in the P2Y12 binding pocket (Figure 5C, dark green dashed lines). The carbonyl oxygen of the peptide bond between the two Hyp residues in OOGGHG formed a hydrogen bond with Cys194 in the P2Y12 binding pocket, while the hydroxyl oxygen on the pyrrolidine ring of the second Hyp interacted with Asn191. The carbonyl oxygen of the Gly–Gly peptide bond formed a hydrogen bond with Lys179. Additionally, the carbonyl oxygen of the peptide bond between the second Gly residue and His established hydrogen bonds with Lys280 and Arg256, whereas the amide NH group of the same peptide bond acted as a hydrogen bond donor to Cys175. The hydroxyl group of the C-terminal carboxyl moiety of OOGGHG also donated a hydrogen bond to Glu281. This interaction pattern is similar to that of the previously reported OG-containing peptides, which also engage key residues such as Lys80, Cys175, Lys179, Asn191, and Lys280 [29]. Hydrophobic interactions may further contribute to binding, with the pyrrolidine ring of the second Hyp residue forming alkyl-type hydrophobic contacts with Tyr105, His187, and Val190 (Figure 5C, pink dashed lines). In addition, the imidazole ring of His participated in π–σ interactions with Leu284 and π–cation interactions with Lys80 (Figure 5C, purple and orange dashed lines). However, an unfavorable donor–donor interaction was observed between this amide NH group and the hydroxyl group of Ser101 (Figure 5C, red dashed lines), suggesting localized steric or electrostatic repulsion that may slightly weaken binding affinity. These results indicate that the second Hyp and His are the major contributors to the interaction between OOGGHG and P2Y12. Specifically, the second Hyp residue contributed to P2Y12 binding through both one hydrogen bond and three hydrophobic interactions. The peptide bond between the second Gly and His formed three hydrogen bonds, while the imidazole ring of His further strengthened the interaction.

Compared with OOGGHG, MTGP was predicted to form only four hydrogen bonds with key residues in the P2Y12 active site (Figure 5F, dark green dashed lines), resulting in a lower absolute binding energy. The carbonyl oxygen of the Met–Thr peptide bond in MTGP established hydrogen bonds with Lys280 and Arg256 of P2Y12. Additionally, the carbonyl oxygen of the Gly–Pro peptide bond and the hydroxyl group of the C-terminal carboxyl moiety in MTGP formed hydrogen bonds with Asn191 of P2Y12. These residues in P2Y12 also formed hydrogen bonds with OOGGHG, suggesting that they are critical for antiplatelet peptide binding. Hydrophobic interactions in MTGP are primarily mediated by the Met and Pro residues, in contrast to OOGGHG, where they are contributed by Hyp. The Met side chain forms alkyl interactions with Lys80 and Phe104 via its hydrophobic thioether group (Figure 5F, pink dashed lines), consistent with previous reports that Met enhances peptide binding stability through hydrophobic interactions [32]. The Pro residue may contribute additional alkyl contacts with Val102 and Val190 (Figure 5F, pink dashed lines), potentially enhancing the stability of the ligand–receptor complex. An unfavorable interaction between the N-terminal amino group of Met and the guanidino group of Arg93 (Figure 5F, red dashed lines), which may slightly weaken the local binding forces. Overall, the relative contributions of the residues to MTGP binding to P2Y12 are suggested to decrease in the order of Pro ≈ Met > Thr > Gly. The Pro residue contributed the most by forming two hydrophobic interactions and one hydrogen bond. The Met residue formed two hydrophobic interactions, while the peptide bond between Met and Thr established two hydrogen bonds.

## 4. Conclusions

In general, hydrolysates of rainbow trout skin collagen could effectively inhibit platelet aggregation induced by ADP in vitro. Peptides MTGP and OOGGHG were the most bioactive components in the hydrolysate, with IC_50_ values of 0.52 mM and 0.54 mM, respectively. This study confirms that OG- or GP-containing peptides might be the main effective peptides in collagen hydrolysate. Molecular docking analysis suggests that MTGP and OOGGHG bind to the active site of the P2Y12 receptor primarily through hydrogen bonds with key residues, including Lys280, Arg256, and Asn191, while hydrophobic interactions involving Hyp in OOGGHG and Met and Pro in MTGP further contribute to the stabilization of the ligand–receptor complex. This study provided scientific support for the development of rainbow trout skin collagen as a provider of natural functional foods. Nevertheless, further experimental validation is needed to elucidate the interaction between the identified peptides and the P2Y12 receptor, as well as the downstream signaling pathways involved. In addition, in vivo studies are required to verify collagen hydrolysate and peptides’ efficacy in animal models and evaluate their bioavailability and safety, thereby providing further evidence for using them as functional food ingredients.

## Figures and Tables

**Table 1:** Amino acid composition of native rainbow trout skin collagen, its collagen hydrolysates, and sub-fraction N2-2.

Amino Acids	Native Skin Collagen g/100 g	Collagen Hydrolysates g/100 g	N2-2 g/100 g
Glu	7.89 ± 0.33	6.50 ± 2.80	6.18 ± 0.20
Asp	4.64 ± 0.46	5.10 ± 0.03	1.20 ± 0.03
Hyp	5.63 ± 1.08	6.90 ± 0.03	13.96 ± 1.31
Ser	4.41 ± 0.06	6.42 ± 2.75	6.95 ± 0.23
Gly	29.74 ± 0.76	30.84 ± 0.60	33.64 ± 1.74
His	0.97 ± 0.07	0.72 ± 0.31	0.30 ± 0.01
Thr	2.27 ± 0.13	2.22 ± 0.04	1.63 ± 0.08
Arg	6.22 ± 0.23	6.10 ± 0.03	3.09 ± 0.21
Ala	8.31 ± 1.28	9.27 ± 0.13	10.24 ± 0.56
Pro	9.71 ± 0.58	10.31 ± 0.04	15.75 ± 0.13
Tyr	0.52 ± 0.01	0.71 ± 0.30	0.07 ± 0.00
Val	2.10 ± 0.02	2.17 ± 0.01	2.02 ± 0.08
Met	1.53 ± 0.08	1.60 ± 0.01	0.49 ± 0.03
Ile	1.15 ± 0.08	1.12 ± 0.01	0.89 ± 0.05
Leu	2.19 ± 0.01	2.24 ± 0.04	1.06 ± 0.05
Phe	1.61 ± 0.04	1.68 ± 0.02	0.56 ± 0.04
Lys	3.88 ± 0.05	3.90 ± 0.03	1.48 ± 0.06
Hydrophilic AAs	42.56 ± 0.51	47.07 ± 1.91	56.21 ± 0.78
Hydrophobic AAs	32.79 ± 1.68	34.47 ± 0.20	34.08 ± 0.96
Total	92.72± 1.84	97.76 ± 0.38	99.74 ±0.48

Note: Hydrophilic amino acids were calculated as the sum of Gly, Ser, Thr, Tyr, and Hyp, while hydrophobic amino acids were calculated as the sum of Ala, Pro, Val, Met, Ile, Leu, and Phe. Asn and Gln were converted to Asp and Glu during acid hydrolysis, respectively. Cys and Trp were not detected.

**Table 2:** Top 20 most abundant oligopeptides identified in N2-2 by LC–MS/MS and their binding energies with the P2Y12 receptor.

Oligopeptides	*m*/*z*	z ^a^	Retention Time	Abundance	−10lgP	Protein Fragment ^b^	Binding Energy ^c^ kcal/mol
GGAQGPAGPT	812.388	1	0.0817	6.48 × 10^8^	37.82	Col I α1a 762-771	−3.0
VGGOGPAG	627.309	1	28.7252	4.22 × 10^8^	28.63	Fib-Col NC1 829-836	−6.5
IAQPAQE	756.388	1	2.2322	2.52 × 10^8^	26.94	Col I α1a 1186-1192	−4.8
AGOQGPAGPS	854.4	1	2.0798	2.27 × 10^8^	23.79	Col XVII α1 931-940	−2.1
AAGPOGAQ	684.33	1	53.786	1.56 × 10^8^	20.77	Col I α1a 909-916	−3.6
IGPOGPTGAH	919.462	1	3.9035	1.47 × 10^8^	28.23	Col I α1b 760-769	2.6
LEGNMG	620.27	1	1.7162	1.04 × 10^8^	38.63	Fib-Col NC1 699-704	−7.1
MTGP	405.18	1	11.848	9.01 × 10^7^	20.78	Col I α1a 750-753	−6.8
VGPOGPSGN	797.38	1	0.0351	8.84 × 10^7^	23.77	Col I α1a 868-876	−2.8
AGPPGGDGQP	852.384	1	5.2127	8.53 × 10^7^	28.63	Col I α1a 799-808	−1.4
NGEMGPA	675.275	1	1.6203	7.05 × 10^7^	31.16	TACC2b 164-1170	−6.1
GPAGPAGQ	654.319	1	53.2253	6.03 × 10^7^	28.49	Fib-Col NC1 788-795	−5.2
ANGPMG	547.218	1	1.7134	5.79 × 10^7^	28.54	Col I α1b 504-509	−8.4
VQGPAGPA	696.367	1	2.4327	5.56 × 10^7^	28.61	Col I α1a 441-448	−5.8
QGPPGPSGQ	824.388	1	1.5737	5.13 × 10^7^	28.54	Col I α1 1102-1110	−3.6
AGPAGPAG	597.298	1	53.7749	5.08 × 10^7^	27.17	Fib-Col NC 259-266 and 787-794	−6.4
GAPGPGGPT	710.347	1	2.0587	4.57 × 10^7^	27.77	Col I α1a 770-778	−3.7
AGPPGAD	584.267	1	39.7301	4.46 × 10^7^	28.62	Col I α1b 805-811	−4.6
OOGGHG	553.225	1	11.8587	4.21 × 10^7^	28.35	Fib-Col NC 706-711	−8.2
VGAPGAOG	641.324	1	1.613	3.67 × 10^7^	28.18	Col I α1a 601-608	−1.9

Note: ^a^, z indicates the charge state of precursor ions. ^b^, Protein fragment annotation was performed via sequence alignment against the Oncorhynchus mykiss protein database in UniProt (https://www.uniprot.org/). The abbreviations and corresponding full names, together with UniProt accession numbers, are shown below: Col I α1a (collagen type I alpha-1a, A0A8C7R225), Fib-Col NC1 (fibrillar collagen NC1 domain-containing protein, A0A060X162), Col XVII α1 (collagen alpha-1(XVII) chain, A0A8L0DRP2), Col I α1b (collagen type I alpha-1b, A0A8L0DR16), TACC2b (tetratricopeptide repeat, ankyrin repeat, and coiled-coil domain-containing protein 2b, A0A8C7W553), and Col I α1 (collagen α1(I), A0A8C7R226). The numbers refer to the amino acid residue positions of identified peptides in the corresponding precursor proteins. ^c^, Binding energies (kcal/mol) represent the molecular docking results between peptides and the P2Y12 receptor obtained using AutoDock Vina.

## Data Availability

The original contributions presented in this study are included in the article. Further inquiries can be directed to the corresponding author.

## Abbreviations

The following abbreviations are used in this manuscript:

Gly	glycine
Pro	proline
Hyp	hydroxyproline
ACE	angiotensin-converting enzyme
CVDs	cardiovascular diseases
P2Y12	purinergic receptor P2Y12
ADP	adenosine diphosphate
DH	degree of hydrolysis
OPA	o-phthaldialdehyde
PRP	platelet-rich plasma
PPP	platelet-poor plasma
ARC	aggregation rate of the control group
ARS	aggregation rate of the sample group
PITC	phenyl isothiocyanate
